# Sub-Optimal Vitamin B-12 Levels among ART-Naïve HIV-Positive Individuals in an Urban Cohort in Uganda

**DOI:** 10.1371/journal.pone.0040072

**Published:** 2012-07-02

**Authors:** Aggrey S. Semeere, Damalie Nakanjako, Henry Ddungu, Andrew Kambugu, Yukari C. Manabe, Robert Colebunders

**Affiliations:** 1 Infectious Diseases Institute, Makerere University College of Health Sciences, Kampala, Uganda; 2 College of Health Sciences, Makerere University, Department of Medicine, Kampala, Uganda; 3 Mulago Hospital, Department of Medicine, Kampala, Uganda; 4 Division of Infectious Diseases, Department of Medicine, Johns Hopkins School of Medicine, Baltimore, Maryland, United States of America; 5 University of Antwerp and Institute of Tropical Medicine, Antwerp, Belgium; Vanderbilt University, United States of America

## Abstract

Malnutrition is common among HIV-infected individuals and is often accompanied by low serum levels of micronutrients. Vitamin B-12 deficiency has been associated with various factors including faster HIV disease progression and CD4 depletion in resource-rich settings. To describe prevalence and factors associated with sub-optimal vitamin B-12 levels among HIV-infected antiretroviral therapy (ART) naïve adults in a resource-poor setting, we performed a cross-sectional study with a retrospective chart review among individuals attending either the Mulago-Mbarara teaching hospitals’ Joint AIDS Program (MJAP) or the Infectious Diseases Institute (IDI) clinics, in Kampala, Uganda. Logistic regression was used to determine factors associated with sub-optimal vitamin B-12. The mean vitamin B-12 level was 384 pg/ml, normal range (200–900). Sub-optimal vitamin B-12 levels (<300 pg/ml) were found in 75/204 (36.8%). Twenty-one of 204 (10.3%) had vitamin B-12 deficiency (<200 pg/ml) while 54/204 (26.5%) had marginal depletion (200–300 pg/ml). Irritable mood was observed more among individuals with sub-optimal vitamin B-12 levels (OR 2.5, 95% CI; 1.1–5.6, *P* = 0.03). Increasing MCV was associated with decreasing serum B-12 category; 86.9 fl (±5.1) vs. 83 fl (±8.4) vs. 82 fl (±8.4) for B-12 deficiency, marginal and normal B-12 categories respectively (test for trend, *P = *0.017). Compared to normal B-12, individuals with vitamin B-12 deficiency had a longer known duration of HIV infection: 42.2 months (±27.1) vs. 29.4 months (±23.8; *P = *0.02). Participants eligible for ART (CD4<350 cells/µl) with sub-optimal B-12 had a higher mean rate of CD4 decline compared to counterparts with normal B-12; 118 (±145) vs. 22 (±115) cells/µl/year, *P = *0.01 respectively. The prevalence of a sub-optimal vitamin B-12 was high in this HIV-infected, ART-naïve adult clinic population in urban Uganda. We recommend prospective studies to further clarify the causal relationships of sub-optimal vitamin B-12, and explore the role of vitamin B-12 supplementation in immune recovery.

## Introduction

Reduced serum micronutrient levels are common among HIV-infected individuals prior to antiretroviral therapy (ART) [1], [2], [3]. Sub-optimal vitamin B-12 has been reported in 10–35% of individuals with HIV infection [3], [4], [5]. A number of factors have also been associated with sub-optimal serum vitamin B-12. Among them are faster HIV disease progression [3], CD4+ T-cell decline [3], [4], [6] anemia [1], weight loss [1], [7], diarrhea [1] neuro-cognitive changes [8], [9] and neuropathy/myelopathy [10].

In order to ascertain the need for micronutrient replacement, World Health Organization (WHO) recommends that the extent and nature of micronutrient depletion among populations of interest be determined [11] since geographical, racial, dietary and cultural differences may influence micronutrient levels especially in low-income countries [12]. Supplemantion with vitamins could be beneficial as noted among HIV-infected pregnant women who received multivitamin supplements which resulted in higher CD4 and CD8 counts, lower viral loads, and a delayed progression to WHO stage III and IV disease [13]. Vitamin B-12 may modulate cellular immunity and has been shown to facilitate production of T lymphocytes, as well as maintain lymphocyte counts within normal range when administered to individuals with pernicious anemia [14].

Although empirical multivitamin supplementation has been shown to improve immune recovery, there is barely any data regarding the burden as well as the clinical and laboratory manifestations associated with vitamin B-12 depletion among individuals receiving HIV/AIDS care in Africa. Currently, vitamin B-12 assay is not a routine pre-ART test in resource-limited settings and there is a paucity of data concerning vitamin B-12 depletion and its role in immune recovery among HIV-infected individuals. We therefore sought to determine the prevalence of sub-optimal serum vitamin B-12 and its associated factors among ambulatory, ART-naïve, HIV-infected adults at two urban clinics in Kampala, Uganda.

## Methods

### Study Design and Selection Criteria

Between March and April 2010 we performed a cross-sectional study with a retrospective chart review among HIV-positive, ART-naïve adults (≥18 years) attending either the Mulago-Mbarara Teaching Hospitals’ Joint AIDS Program (MJAP) or the Infectious Diseases Institute (IDI) clinic. Due to the increased physiological demand for vitamin B-12 in pregnancy, we excluded pregnant women.

### Study Setting

Both the MJAP and IDI clinics are located within the Mulago Hospital Complex and offer comprehensive care to HIV-infected adults within the hospital’s catchment area. IDI offers free outpatient HIV/AIDS care and has registered over 25,000 patients since its inception in 2002, with about 10,000 actively in care, 7,000 of whom have been initiated on ART. The MJAP clinic is part of a collaborative partnership between Makerere University College of Health Sciences, Mbarara University Faculty of Medicine with Mulago and Mbarara teaching hospitals, respectively. Currently over 8000 patients are registered and in care at the MJAP clinic. Both MJAP and IDI clinics offer ART-naïve individuals monthly cotrimoxazole prophylaxis and biannual CD4 tests. Serum vitamin B-12 assessment is not part of routine care and multivitamins are only prescribed at the clinicians’ discretion.

### Data Collection

From a list of registered ART-naïve participants attending the clinic, we selected every 10^th^ participant until the sample size was attained. For every participant that did not fulfill the inclusion criteria, the next participant was approached to join in the study. Selected participants were then reviewed by the investigator and requested to provide written informed consent in either Luganda or English (depending on language of preference). The study questionnaire was then administered together with a clinical examination to obtain clinical parameters. The participants’ chart were also reviewed for the date of HIV diagnosis, CD4 count at diagnosis, history of opportunistic infections, WHO disease stage and prescription records for vitamin B-12 containing supplements. Participants’ weight and height were measured using a calibrated weight scale and height board respectively. Six milliliters (mls) of blood were drawn by a laboratory technician of which 3 mls was used for complete blood cell count (CBC) and peripheral film, and the other 3 mls for serum vitamin B-12 assay. For participants that did not have a CD4 count within 3 months prior to study visit, an extra 3 ml of blood was drawn for CD4 count estimation. Samples were delivered to a laboratory daily within six hours of collection. Subjects found with vitamin B-12 deficiency (<200 pg/ml) received 3 doses of 1 mg parenteral vitamin B-12 in addition to the nutritional counseling while those marginal B-12 (200–300 pg/ml) only got nutritional counseling.

### Laboratory Assays

CBC was measured at the Makerere University-John’s Hopkins University (MUJHU) core laboratory at the IDI, which maintains accreditation by the College of American Pathologists. A thin film, stained with Giemsa, was used to study the blood cell morphology. CD4 counts were estimated by flow cytometry from either the IDI or MJAP laboratories. Vitamin B-12 levels were measured using the Roche diagnostics Elecsys 2010© immunoassay at Ebenezer clinical laboratories, a Kampala laboratory accredited by the South African National Accreditation System (SANAS) and the International Organization for Standardization (ISO).

### Ethics Statement

Ethical approval for this study was obtained from the Institutional Review Board (IRB) of the College of Health Sciences, Makerere University and the Scientific Review Committee of the Infectious Diseases Institute. All participants provided written informed consent.

### Statistical Analysis

Data were entered into an Epidata© database before being exported and analyzed in STATA© version 12 (STATA Corp, College Station, TX) software. Continuous variables were presented as either medians or means depending on the distribution of the data. We used the Student t-test to compare means and the Mann-Whitney U test to compare medians. Categorical variables were analyzed and presented as frequencies and percentages; they were compared using the chi-square and Fisher’s exact tests as appropriate. The prevalence of sub-optimal serum vitamin B-12 was expressed as the proportion of participants with sub-optimal serum vitamin B-12 over the total number of participants with a vitamin B-12 result. The main outcome of this study was sub-optimal vitamin B-12 serum level defined as a value below 300 pg/ml (221 pmol/L) [5], [11]. Other outcomes evaluated included vitamin B-12 deficiency defined as a level less than 200 pg/ml (148 pmol/L) and marginal B-12 depletion defined as a level between 200–300 pg/ml (148–221 pmol/L) [5], [11]. Change in CD4 count was the difference between CD4 count at the time of B-12 assessment (current CD4 count) and the CD4 count at HIV diagnosis. The rate of change in CD4 count was derived from the change in CD4 count and the time between HIV diagnosis date and study date (B-12 assessment date). We defined anemia as hemoglobin level <10 g/dl. Bivariate analysis was done to determine association between sub-optimal vitamin B-12 and other factors.

We used multivariate logistic regression to determine the independent association between predictors and sub-optimal B-12 deemed appropriate based on prior published work and biologic plausibility. From our substantive knowledge of the possible causal relationships, we generated Directed Acyclic Graphs (DAGs) to best define the relationships first. We then used multivariate logistic models based on these DAGs and we present both adjusted and unadjusted estimated odds ratios for relationships between the predictors we studied and sub-optimal vitamin B-12. Among the predictors, age, gender, occupation, WHO disease stage, body mass Index (BMI),CD4 count at HIV diagnosis and current CD4 count, CD4 count change, rate of change in CD4, known duration of HIV infection, anemia, MCV, dietary intake and clinical factors including “irritable mood” were studied. We used the Hosmer-Lemeshow test to rule out gross lack of fit in the final models we present. We repeated multivariate models in two sensitivity analyses; the first for participants who had not taken vitamin B-12 containing supplements, and a second for participants eligible for ART-based on a CD4 cut-off of <350 cells/µL.

## Results

Of the 210 participants screened, 152 were from IDI and 58 from MJAP. Out these, five were excluded (three were pregnant, two were too ill to give written informed consent) and one did not have blood drawn. We present data for 204 enrolled participants. The mean age of the study participants was 34.4 (±9.4) years with 72% of them females−consistent with the demographic distribution of patients at both clinics. The mean serum vitamin B-12 level for all study participants was 384.6 pg/ml (±197.7) while the median B-12 level was 349 pg/ml [Inter Quartile Range (IQR) 254.2–450.5 pg/ml].

Seventy-five (36.8%) participants had sub-optimal serum vitamin B-12 (<300 pg/ml) with 21 (10.3%) being vitamin B-12 deficient (<200 pg/ml) and 54 (26.5%) with marginal vitamin B-12 depletion (200 to 300 pg/ml). Prevalence of sub-optimal B-12 did not vary by study site (Table 1). The distribution of characteristics such as age, gender, level of education, WHO stage and opportunistic infections and/or HIV-associated disease conditions were largely similar for both normal and sub-optimal B-12 categories (Table 1). The proportion of participants with irritable mood however, was higher among participants with sub-optimal vitamin B-12 compared to those with normal vitamin B-12 levels (25.3% vs. 13.3%; *P = *0.03) (Table 1). Anemia, leucopenia and thrombocytopenia were not associated with sub-optimal vitamin B-12 level but mean MCV for participants with B-12 deficiency was higher for sub-optimal B-12 compared to normal B-12 though this was not statistically significant (Table 1). Compared to marginal and normal B-12 levels however, individuals with B-12 deficiency had higher mean MCV: 83 fl (±8.4) vs. 82 fl (±8.4) 86.9 fl (±5.1) respectively. This trend of increasing MCV across decreasing serum B-12 category was statistically significant (test for trend, *P = *0.017). Five participants had anemia (hemoglobin <10 g/dl) and sub-optimal vitamin B-12. One of these had B-12 deficiency while the other two had marginal depletion. Two of the five participants with anemia also had a mean corpuscular volume (MCV) >100fl.

Known duration of HIV infection was slightly longer for individuals with sub-optimal vitamin B-12 though this difference was not statistically significant (Table 1). Considering B-12 deficiency, marginal depletion and normal B-12 categories, we found a statistically significant association in the trend of increasing mean duration of known HIV infection with decreasing B-12 category: with normal B-12 at 29.4 months (±23.8), marginal B-12 at 33.6 (±30.0) and B-12 deficiency 42.2 months (±27.1) (test for trend, *P = *0.03).

**Table 1:** A comparison of characteristics by serum vitamin B-12 group among adult HIV-infected ART naïve participants at two urban HIV clinics in Uganda, in April 2010.

Characteristic	Category	Sub-optimal Vitamin B-12(<300 pg/ml) N = 75	Normal VitaminB-12 (>300 pg/ml) N = 129	P-value
**Age (years), N (%)**	*<29*	24(32.0)	49(38.3)	0.66
	*30–49*	46(61.3)	70(54.7)	
	*>50*	5 (6.7)	9(7.0)	
**Female, N (%)**		57 (76.0)	90 (69.8)	0.34
**Married, N (%)**		41(54.7)	67(51.9)	0.71
**Education, N (%)**	*No Education*	6 (8.0)	15 (11.6)	0.26
	*Basic Education*	28 (37.3)	59 (45.7)	
	*Secondary/Tertiary*	41 (54.7)	55 (42.6)	
**Occupation**	*Unemployed*	13(17.3)	35(26.9)	0.12
	*Employed*	62(82.7)	95(73.1)	
**Study Site, N (%)**	*IDI*	56 (37.8)	92 (62.2)	0.55
	*MJAP*	19 (33.3)	38 (66.7)	
**Use of B-12 Supplement, N (%)**		32(42.7)	45(34.9)	0.27
**Alcohol intake, N (%)**		26(34.7)	54(42.2)	0.29
**WHO Staten (%)**	*WHO Stage I & II*	64 (85.3)	108 (83.7)	
	*WHO Stage III & IV*	11 (14.7)	21 (16.3)	0.76
**Irritable mood, N (%)**		19 (25.3)	17 (13.2)	**0.03**
**Paraesthesia, N (%)**		2 (2.7)	5 (3.9)	0.65
**Opportunistic Infections, N (%)**	*Tuberculosis*	7(9.3)	12(9.4)	0.99
	*Oral candidiasis*	20(26.7)	39(30.5)	0.56
	*Herpes zoster*	15(20.0)	18(14.1)	0.27
	*Genital sores*	14(18.7)	30(23.4)	0.43
	*Recurrent Pneumonia*	2(2.7)	10(7.8)	0.14
	*Skin rashes(PPE)*	30(40.0)	68(53.1)	0.07
	*Diarrhea*	5(3.9)	5(3.9)	1.00
**Anemia, N (%)**		5 (6.7)	18 (13.9)	0.11
**BMI, mean (SD)** a		24.7(4.7)	23.4(4.6)	0.07
**MCV ** ***(fl),*** ** mean (SD)**		84.0(7.8)	82.0(8.4)	0.08
**CD4 at diagnosis ** ***(cells/µl )*** ** median (IQR)** a		542 (410–714)	501(370–662)	0.11
**Current CD4 ** ***(cells/µl )*** ** median (IQR)**		406 (326–607)	418 (314–547)	0.71
**CD4<350 ** ***(cells/µl )*** ** N (%)**		25 (33.3)	40 (30.8)	0.70
**Known duration with HIV ** ***(months),*** ** mean (SD)** a		36.1 (30.0)	29.3(23.8)	0.09

**IQR-** Inter Quartile Range, **BMI**- Body Mass Index, **WHO**-World health Organization, **MCV**-Mean Corpuscular Volume, **Hb**- Hemoglobin. **IDI**- Infectious Disease Institute, **MJAP**- Mulago-Mbarara Teaching Hospitals’ Joint AIDS Program T-tests were used to compare means and the chi-square for proportions, except where mentioned.

a Some missing data. N = 113 for Normal B-12 & 70 for Sub-Optimal B-12.

Use of vitamin B-12 containing supplements did not vary between the B-12 categories. Further, all participants surveyed reported taking animal source foods as part of their diet. No participant reported being a strict vegetarian. Only 5.8% of participants reported having taken animal source foods less than once in the previous year and this did not vary with serum vitamin B-12 category. Reported use of alcohol was not different between the groups. Use of proton pump inhibitors and H_2_ receptor antagonists was 2.5% and 3.5% respectively and none of the participants surveyed reported use of metformin.

Adjusted for age, gender, BMI, supplement use, occupation, known duration with HIV and current CD4 with multivariate logistic regression, sub-optimal B-12 was associated with irritable mood, OR 2.5 (95% CI; 1.1–5.6, *P* = 0.03) (Table 2). Female sex, anemia, WHO stage, being employed and supplement use were associated with sub-optimal B-12 but these findings were inconclusive based on the corresponding p-value and 95% CI for the odds ratios (Table 2).

**Table 2:** Risk factors for sub-optimal vitamin B-12 among adult HIV-infected ART naïve individuals at two urban HIV clinics in Uganda, in April 2010.

Predictor variable	Unadjusted OR	95% CI	*P*-value	Adjusted OR	95% CI	*P*-value
**Age**	1.0	0.9–1.04	0.42	1.0^a^	0.9–1.0	0.75
**Female**	1.4	0.7–2.7	0.30	1.7^a^	0.8–3.9	0.19
**Irritable Mood**	**2.2**	**1.1–4.6**	**0.03**	**2.5** a	**1.1–5.6**	**0.03**
**Supplement use**	0.7	0.4–1.3	0.25	0.7^a^	0.4–1.3	0.30
**WHO Stage III & IV**	0.8	0.4–1.9	0.7	1.0^a^	0.4–2.4	0.93
**Occupation (Employed)**	1.7	0.8–3.6	0.13	1.7^a^	0.7–4.0	0.21
**Current CD4**	1.0	0.9–1.0	0.90	1.0^a^	0.9–1.0	0.50
**BMI**	1.1	0.9–1.2	0.07	1.0^a^	0.9–1.1	0.30
**Known duration with HIV**	1.1	1.0–1.3	0.08	1.0^a^	1.0–1.1	0.24
**Anemia**	0.4	0.1–1.6	0.21	0.4^a^	0.2–1.5	0.20
**MCV**	1.0	0.9–1.1	0.08	1.0^a^	0.9–1.1	0.50
**Rate of CD4 decline**	1.0	0.9–1.0	0.32	1.0^b^	0.9–1.0	0.24

a Adjusted for age, sex, BMI, supplement use, MCV, occupation, irritable mood, known duration with HIV, WHO disease stage and current CD4.

b Adjusted for,age, sex, BMI, WHO stage, supplement use.

**BMI**- Body Mass Index, **MCV**-Mean Corpuscular Volume, **OR** Odds Ratio, **WHO**-World health Organization.

In the sensitivity analysis for participants without history of B-12 supplementation we found statistically significant adjusted odds ratios for the association between irritable mood and sub-optimal B-12 at 3.2 (95% CI: 1.1–9.3, *P* = 0.04) (Table 3). Average MCV was also significantly higher among the participants with a sub-optimal B-12 level at 85.0 fl (±7.5), compared to those with normal B-12 at 81.0 fl (±8.4), *P = *0.02. The adjusted odds ratios for this association were marginally statistically significant; 1.1 (95%CI; 1.0–1.1, *P* = 0.03) (Table 3). The mean duration of known HIV infection, was also statistically significantly higher among participants with sub-optimal B-12 compared to those with normal B-12 at 37 (±28.0) vs. 26 (±22.4) months (*P = *0.017), respectively. This association remained marginally significant with multivariate logistic regression; OR 1.0 (95%CI; 1.0–1.1, *P* = 0.02) (Table 3). Participants with sub-optimal B-12 had significantly higher mean BMI, 25.4 Kg/m^2^ (±5.2) compared to those with normal B-12, 23.0 Kg/m^2^ (±4.2) *P* = 0.006. Adjusted for age, sex, occupation, current CD4, and known duration with HIV, BMI had just marginal statistical association with sub-optimal B-12, OR 1.1 (95%CI; 1.0–1.3, *P* = 0.02) (Table 3). For individuals who reported use of supplements, sub-optimal B-12 was associated with female sex OR 5.2 (95%CI; 1.3–20.4, *P* = 0.02).

**Table 3:** Risk factors for sub-optimal serum vitamin B-12 among adult HIV-infected ART naïve participants who did not report using vitamin B-12 containing supplements at two urban HIV clinics in Uganda, in April 2010.

Predictor variable	Unadjusted OR	95% CI	*P*-value	Adjusted OR	95% CI	*P*-value
**Age**	1.0	0.9–1.04	0.9	0.9^a^	0.8–1.0	0.09
**Female**	1.1	0.5–2.5	0.70	0.6^a^	0.2–2.1	0.47
**Irritable Mood**	**2.6**	**1.1–6.4**	**0.03**	**3.2** a	**1.1–9.3**	**0.04**
**WHO Stage III & IV**	1.9	0.4–2.5	0.90	1.9^a^	0.6–6.2	0.30
**Occupation (Employed)**	1.9	0.7–4.5	0.20	1.9^a^	0.6–6.1	0.27
**Current CD4**	1.0	0.9–1.0	0.80	1.0^a^	0.9–1.0	0.90
**BMI**	**1.1**	**1.0–1.2**	**0.01**	**1.1** a	**1.0–1.3**	**0.01**
**Known duration with HIV**	**1.2**	**1.0–1.5**	**0.02**	**1.0** a	**1.0–1.1**	**0.02**
**Anemia**	0.6	0.2–2.5	0.50	0.6^a^	0.1–2.6	0.40
**MCV**	**1.1**	**1.0–1.1**	**0.02**	**1.1** a	**1.0–1.2**	**0.02**
**Rate of CD4 decline**	1.0	0.9–1.0	0.82	1.0^b^	0.9–1.0	0.46

a Adjusted for age, sex, BMI, supplement use, MCV, occupation, irritable mood, known duration with HIV, WHO disease stage and current CD4.

b Adjusted for,age, sex, BMI, WHO stage, supplement use.

**BMI**- Body Mass Index, **MCV**-Mean Corpuscular Volume, **OR** Odds Ratio, **WHO**-World health Organization.

Among participants eligible for ART (CD4≤350 cells/µl) at time of study, mean rate of CD4 decline was significantly higher for patients with sub-optimal B-12 at 118 (±145) compared to 22 (±115) cells/µl/year, *P = *0.01, for patients with normal B-12 levels. This association was marginally significant with multivariate analysis OR 1.0 (95%CI; 0.9–1.1, *P* = 0.02) (Table 4). In this group however, irritable mood was strongly associated with sub-optimal B-12: OR 8.6 (95%CI; 2.0–37.0, *P* = 0.004) (Table 4). We found no associations with sub-optimal vitamin B-12 levels among individuals with CD4>350 cells/µl.

**Table 4:** Risk factors for sub-optimal B-12 among adult HIV- infected ART naïve participants eligible for ART (CD4<350 cells/µl) at two urban HIV clinics in Uganda, in April 2010.

Predictor variables	Unadjusted OR	95% CI	*P*-value	Adjusted OR	95% CI	*P*-value
**Age**	1.0	0.9–1.0	0.50	1.0^a^	0.9–1.1	0.24
**Female**	1.5	0.5–4.7	0.50	1.5^a^	0.3–7.3	0.60
**Irritable Mood**	**5.2**	**1.6–16.8**	**0.006**	**8.6** a	**2.0–37.0**	**0.004**
**WHO Stage III & IV**	0.8	0.2–2.5	0.60	0.5^a^	0.1–2.6	0.40
**Occupation (Employed)**	1.0	0.3–3.4	0.90	0.5^a^	0.1–2.5	0.40
**Current CD4**	1.0	0.9–1.0	0.90	1.0^a^	0.9–1.1	0.80
**Supplement use**	1.3	0.4–3.7	0.60	2.1^a^	0.5–7.9	0.30
**BMI**	1.0	0.9–1.10	0.90	0.8^a^	0.8–1.1	0.60
**Known duration with HIV**	1.1	0.8–1.30	0.70	1.0^a^	1.0–1.3	0.10
**Anemia**	0.6	0.2–3.4	0.60	0.6^a^	0.1–3.9	0.60
**MCV**	1.0	1.0–1.10	0.30	1.0^a^	1.0–1.1	0.30
**Rate of CD4 decline**	**1.0**	**0.9**–**1.0**	**0.03**	**1.0** b	**0.9**–**1.1**	**0.02**

a Adjusted for age, sex, BMI, supplement use, MCV, occupation, irritable mood, known duration with HIV, WHO disease stage and current CD4.

b Adjusted for,age, sex, BMI, WHO stage, supplement use.

**BMI**- Body Mass Index, **MCV**-Mean Corpuscular Volume, **OR** Odds Ratio, **WHO**-World health Organization.

## Discussion

We found a high prevalence (36.8%) of sub-optimal serum vitamin B-12 (<300 pg/ml) in this ambulatory, urban, HIV-infected, ART-naïve population. Presently, we have limited published data on serum vitamin B-12 levels among populations in sub-Saharan Africa to make direct comparisions with. Considering other work from the region, a study among school children in rural Kenya found 30.5% of them B-12 deficient (<200 pg/ml) while 37.7% had marginal B-12 levels (200–300 pg/ml) [15]. Another study among pregnant Sidama women in southern Ethiopia revealed that 23% had vitamin B-12<150 pmol (<211 pg/ml) [16]. These two studies uniquely highlight the high prevalence of sub-optimal vitamin B-12 depletion among rural populations in Africa. Importantly though, the HIV status of participants in these two above studies was not assessed. Putting our findings in perspective, the HIV-infected, ART-naïve individuals in this study had a lower mean vitamin B-12 level (384 pg/ml) than the mean B-12 level reported in a population of healthy, young, Ugandan, university students (469 pg/ml) [17]. Nontheless there is still a need to evaluate vitamin B-12 levels among rural HIV-infected populations in subsaharan Africa since these individuals are prone to higher levels of poverty that which threatens food security by significantly reducing availability and quality of their nutrition.

Studies from resource-rich settings also corroborate the sub-opitmal B-12 levels among HIV-infected individuals comapred to their HIV-uninfected counter parts [3], [4], [6], [9]. Addtionally, there is also a wide variation in the prevalence estimates of B-12 depletion among HIV-infected individuals in these studies [3], [4], [6], [9], [10], [18], [19]. This is possibily due to the corresponding wide variability in B-12 cut-off values and patient populations used. At a cut-off value of <200 pg/ml, 10.3% of the ART naïve participants in the present study were vitamin B-12 deficient. Similarly, among individuals in developed countries the prevalence of B-12 deficiency ranged between 12 and 13% at comparable cut-off values. In a retrospective study among homosexual and heterosexual men in the Baltimore-Washington DC area, the prevalence of vitamin B-12 deficiency (<120 pmol/l, equivalent to 163 pg/ml) was 12% [19] while Hepburn et al. in Houston Texas, reported a prevalence of 13.1% using a cut-off value of 211 pg/ml [4]. Among 200 HIV-positive individuals in Montreal, Canada, a higher prevalence of 30.5% at a higher cut-off of <300 pmol/l (equivalent to 406 pg/ml) was reported [6]. We note that in some of the aforementioned studies, individuals had taken antiretroviral therapy in varying degrees [4], [5], [8], [9], [16], [20] making their findings not directly comparable to ours. Even so, the 36.8% prevalence of sub-optimal B-12 we find in our study is still high given the 37.5% reported use of vitamin B-12 containing supplements overall.

We found that sub-optimal vitamin B-12 was associated with an “irritable mood”. This finding has not been reported before although other neuro-cognitive disorders have been associated with sub-optimal vitamin B-12 such as; myelopathy [10], neuropathy [10], cognitive changes [8], [9] among HIV-infected adults [20]. In fact, mood disorders such as bipolar affective disorders have been reported among HIV-infected individuals even in Uganda [21], [22]. On the other hand, multivitamin supplementation (B-complex, C and E) has been shown to be protective from depression with a 22% reduction in risk (*P = *0.005) in a randomized control trial among HIV-positive pregnant women in Tanzania [23]. A detailed neuropsychiatric assessment was not possible in this study, therefore more in-depth neuropsychiatric assessment and prospective studies are required to further understand the association between sub-optimal vitamin B-12, irritable mood and affective disorders among HIV-infected adults with and without antiretroviral therapy.

We observed that the participants with sub-optimal vitamin B-12 had higher MCVs compared to those with normal vitamin B-12 levels. This association was still statistically significant with multivariate analysis after eliminating individuals reported to have taken B-12 containing supplementation. Macrocytosis has been observed in previous studies among individuals with sub-optimal vitamin B-12 [24]. Moreover, previous studies have reported that participants with low vitamin B-12 were also more likely to have clinically advanced HIV disease with weight loss, diarrhea and anemia [1], [4], [6], [7], [19], [25]. Individuals in the present study did not have advanced HIV disease since we studied ART naïve patients who were probably slow long-term non-progressors with relatively high CD4 counts and hence less advanced disease.

In addition, low BMI had been associated with sub-optimal B-12 usually among very ill individuals with clinically advanced disease [6]. Surprisingly, we found that participants with sub-optimal B-12 levels had on average a higher BMI. Nutritional assessment in the present study, which not only included measurement of the BMI but also a dietary history, did not reveal any association between low nutritional status and sub-optimal B-12 levels as reported prior. The number of participants with sub-optimal vitamin B-12 and clinical malnutrition was also low. This may in part have resulted from improved HIV patient care at both urban study sites where nutritional counseling and sometimes free nutritionally enhanced food are provided. We cannot however fully explain the association between higher BMI and sub-optimal B-12 that we find and further evaluation is warranted preferably with a prospective study design.

Individuals with sub-optimal vitamin B-12 levels in this study also had a longer known duration of HIV infection especially when those who did not report intake of supplements were considered. This finding may suggest that the depletion of vitamin B-12 may result from progression of HIV disease as noted elsewhere [4], [6], [19], [25]. The higher rate of CD4 decline noted among patients with sub-optimal vitamin B-12 levels with CD4 cells <350, may also suggest that the depletion of vitamin B-12 may result from progression of HIV disease [4], [6], [19], [25]. The reduced vitamin B-12 stores in both cases possibly resulting from increased demand to replace CD4 cells depleted by actively replicating HIV virus. In the present study, we did not measure baseline B-12 (at the time of HIV diagnosis) so this theory is impossible to prove. Further evaluation of the effect of HIV disease progression and recovery on sub-optimal vitamin B-12 and vice versa are thus warranted. Understanding this relationship would be valuable in ascertaining the short and long-term effects of vitamin B-12 replacement as an adjuvant to ART.

There were some limitations in the present study that should be considered while interpreting our findings. When adjusting for measures of social economic status as a determinant of food security in our models, we used a proxy measure (occupation) and this may have been inadequate to fully account for the role social economic status plays in vitamin B-12 depletion. In addition, our results may not be easily generalized to HIV-infected rural populations that have a relatively different risk profile for the development of sub-optimal vitamin B-12. With the present study design, reverse causality cannot also be ruled out for the factors we found associated with sub-optimal vitamin B-12.

We found a high prevalence of vitamin B-12 depletion among ambulatory HIV-infected, ART-naïve adults attending HIV/AIDS care in an urban setting in Uganda. Sub-optimal B-12 was associated with longer duration of HIV infection, higher MCV, irritable mood and higher rate of CD4 decline among individuals eligible for ART. We recommend prospective studies to further clarify the role of vitamin B-12 in HIV disease progression and immune recovery in both rural and urban populations. Individuals with HIV and mood disorders may benefit from assessment of serum vitamin B-12 level.
